# Intraosseous ganglion in the subchondral region of the lateral femoral condyle in an 11-year-old girl: a case report

**DOI:** 10.1186/1758-2555-1-25

**Published:** 2009-11-05

**Authors:** Hiroshi Nakayama, Masayoshi Yagi, Shinichi Yoshiya

**Affiliations:** 1 Department of Orthopaedic Surgery, Hyogo College of Medicine, Nishinomiya, Hyogo, Japan

## Abstract

We report the case of a patient with intraosseous ganglion in the lateral femoral condyle. An 11-year-old girl presented with right knee pain following a twisting injury. Plain radiographs of the knee showed a small circumscribed radiolucency with a thin sclerotic margin in the subchondral region of the lateral femoral condyle. Although the image findings and location are not typical, the lesion was tentatively diagnosed as osteochodritis dissecans. Six months after the conservative treatment with a break from vigorous sports activities, the size of the bony lesion had not decreased. Thus, we performed arthroscopy to make a definitive diagnosis. Arthroscopic examination revealed an area with dimple and surface irregularity at the lateral femoral condyle. On excision of the overlying tissue, the lesion was cystic containing brown mucous fluid. No association between the cyst and the articular structures was observed. Histologic examination of the resected cyst wall showed dense fibrous tissue with spotty areas of calcification. Base on these findings, we made a diagnosis of intraosseous ganglion. At the nine-month postoperative follow-up, the radiographic examination showed healing of the lesion. We speculate that the lesion in this case might have occurred as a result of repetitive overstress or microtrauma.

## Introduction

Intraosseous ganglion is a benign non-neoplastic lesion of unknown etiology. The femoral head and the tibia have been shown to be relatively commonly affected [1]. However, occurrence in the subchondral region of the knee is rare and occurrence in childhood even rarer. We report a case of an 11-year-old girl with an intraosseous ganglion cyst at the subchodral area of the lateral femoral condyle, without communication with the joint. Previously, only two cases in children with similar clinical presentations have been reported.

## Case report

An 11-year-old girl was evaluated for right knee pain following a twisting injury. She belonged to a volleyball club and practiced almost every day. However, no history of antecedent trauma and no relevant medical history were noted. She had suffered mild knee pain in activities of daily living for three days before the injury and been managed by her primary care physician. Radiographic examination was performed by the initial doctor, and she was referred to our clinic for investigation of an abnormal lesion found in the radiograph.

**Figure 1:**
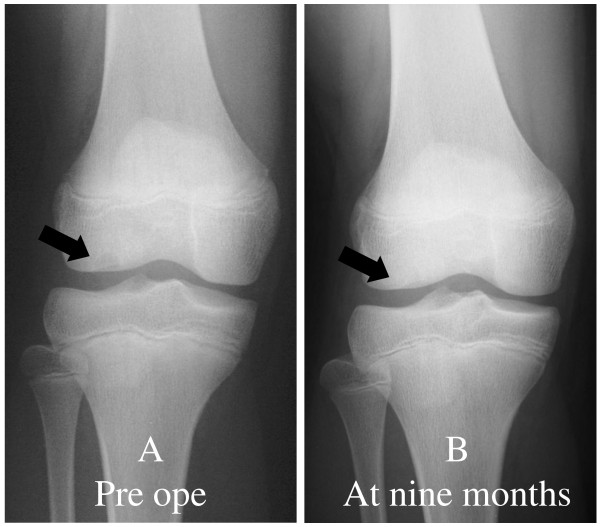
**Pre- and postoperative radiographs**. (A) Preoperative radiograph showing a small circumscribed radiolucency with a thin sclerotic margin in the subchondral region of the lateral femoral condyle (black arrow). (B) Radiograph taken at nine months showing healing of the lesion.

**Figure 2:**
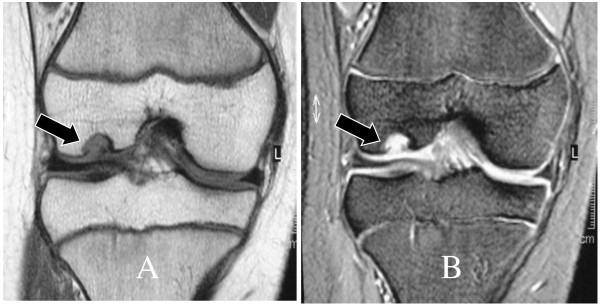
**(A) T1-weighted coronal MR image (B) T2-weighted coronal MR image**. A cystic structure is seen in the lateral femoral condyle (black arrow). A lesion of low signal intensity on T1-weighted image and high signal intensity on T2-weighted image is evident

Physical examination on initial presentation at our clinic showed full range of motion of the affected knee without limping, and effusion, swelling, local heat, instability or tenderness were not detected. Plain radiographs of the knee showed a small circumscribed radiolucency with a thin sclerotic margin in the subchondral region of the lateral femoral condyle. The lesion was located near the contact area at maximum knee extension (Fig 1). The size of the cyst was about seven millimeters in diameter. MRI examination showed a fluid signal in this structure with low signal intensity on T1-weighted images and high signal intensity on T2-weighted images (Fig 2). Complete blood count, erythrocyte sedimentation rate, and serum chemistry studies were normal. In the differential diagnosis, osteochondritis dissecans and tumorous lesions such as chondroblastoma, chondromixoid fibroma, giant cell tumor, and fibrous dysplasia were considered. Based on the patient's age and the radiologic finding of subchondral radiolucency, the lesion was tentatively diagnosed as osteochodritis dissecans. However, its location was atypical and the MRI findings were not coincident with this preoperative diagnosis. Since accompanying clinical symptoms and signs were minimal, we instructed the patient to stop sports activities and wear a hinged brace limiting full extension during the daytime. Six months after starting the treatment, although she remained symptom free, the size of the bony lesion had not decreased, and we decided to perform arthroscopy to make a definitive diagnosis.

**Figure 3:**
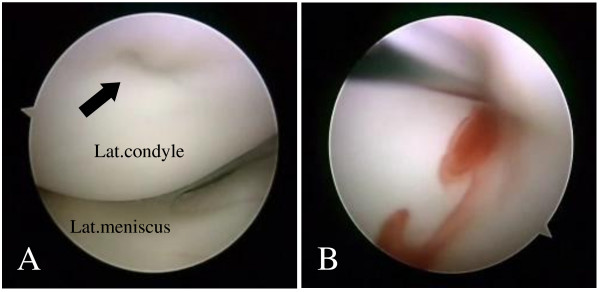
**On arthroscopic examination.** (A) a small dimple and surface irregularity of the overlying cartilage is seen at the lateral femoral condyle, where it contacts at full-extension (arrow). (B) After the curettage, bloody mucous content flows inside the cavity.

**Figure 4:**
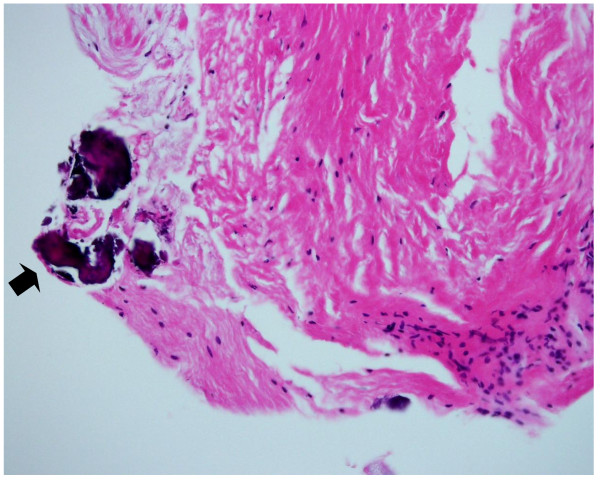
**Photomicrograph of the cyst wall**. The lining tissue consists of dense fibrous tissue with spotty areas of calcification (black arrow) (Hematoxylin and Eosin, ×200).

On arthroscopic examination, an area with a dimple and irregularity was seen at the weight bearing region of the lateral femoral condyle, where it contacted the tibial articular surface at full extension (Fig 3). The remaining structure within the knee joint was normal. On excision of the overlying tissue, the lesion was cystic containing brown mucous fluid. Removal of the surrounding lining tissue and curettage were performed. The biopsy included the wall of the cyst and the overlying cartilage. No association between the cyst and the articular structures such as the joint capsule, cruciate ligaments and meniscusi was observed. Histologically, the lining tissue consisted of dense fibrous tissue with spotty areas of calcification without a continuous synovial layer (Fig 4). These findings were consistent with those reported in previous studies as ganglion [2,3].

The postoperative course was uneventful and the patient was permitted to resume sports activity at 4 months. At the nine-month postoperative follow-up, she remained asymptomatic and the radiographic examination showed the bony lesion was barely identifiable with apparent healing (Fig 1).

## Discussion

Our patient presented with an intraosseous ganglion in the subchondral region of the femoral condyle. The occurrence of a ganglion in this location and age group is rare. The youngest patient previously reported with this type of ganglion was a 6-year-old boy [2]. His past medical history was significant as he suffered from Pierre-Robin syndrome. No ligamentous attachment was seen in his knee, but the cystic lesion existed within the joint. The only other report of an intraosseous ganglion in a younger patient was a 12-year-old boy [4]. In this case, the cyst originated from a posterior cruciate ligament.

The cause of the intrasseous ganglion is unknown. Kambolis reported that ganglionic cysts of the bone developed by invasion of the ganglion-like connective tissue into the bone from the local soft parts [3]. Schajowicz et al. stated that approximately 85% of the reported cases are of intraosseous origin related to altered mechanical stress leading to intramedullary vascular disturbance and aseptic necrosis. They attributed the etiology of the cystic formation in this type to revitalization of the necrotic areas by fibroblastic proliferation and subsequent mucoid degeneration [5].

In the present case, extension of the ganglion into the joint cavity was not seen and the lesion was located in the subchondral region of the lateral femoral condyle. The location of the cyst was in the area of contact where repetitive mechanical stress may be applied at full extension. Since the patient was a volleyball player, we speculated the lesion in this case might have occurred as a result of repetitive overstress or microtrauma. Histological findings of the cyst wall seem to be coincident with our speculation. Arthroscopic examination of the articular surface of the lesion showed a dimple and thinning of the overlying cartilage. There may have been a subtle communication between the cyst and the intraarticular space. The presence of communication between the cyst and the joint has been observed in previously reported cases, and Schajowicz et al. suggested the possibility of subsequent obliteration of the connecting channel [5]. The dimple of the articular surface observed in this case may have been part of a sequence of incomplete obliteration of the entrance of the channel. Another possible etiology of the present case, based on the patient's age and activity, is osteochondritis dissecans of atypical presentation. Although both arthroscopic and histological findings in this case are not consistent with those of osteochondritis dissecdans, repetitive overstress to the weak subchondral bone in a growing athlete is thought to be a common causative factor.

Management of the intraosseous ganglion in previous reports was usually simple curettage with or without bone graft, and the prognosis generally good without recurrence. Only curettage was performed in our case because the lesion was small and left open. Subsequent healing was observed in the following few months. However, continued follow-up is necessary to guard against the possibility of recurrence of the lesion.

## Competing interests

The authors declare that they have no competing interests.

## Authors' contributions

All authors co-wrote the paper and discussed the results and commented on the manuscript. All authors read and approve the final manuscript.
